# Sentiment analysis of the Hamas-Israel war on YouTube comments using deep learning

**DOI:** 10.1038/s41598-024-63367-3

**Published:** 2024-06-13

**Authors:** Ashagrew Liyih, Shegaw Anagaw, Minichel Yibeyin, Yitayal Tehone

**Affiliations:** 1 Department of Software Engineering, Debre Markos University, Debre Markos, Ethiopia; 2 Department of Business, University of Southeastern Norway, Drammen, Norway; 3 Department of Information Technology, Debre Markos University, Debre Markos, Ethiopia

**Keywords:** Deep learning approach, Recurrent neural network, Sentiment analysis, Word2vec, FastText, GloVe, Mathematics and computing, Computer science, Information technology

## Abstract

Sentiment analysis aims to classify text based on the opinion or mentality expressed in a situation, which can be positive, negative, or neutral. Therefore, in the world, a lot of opinions are available on various social media sites, which must be gathered and analyzed to assess the general public’s opinion. Finding and monitoring comments, as well as manually extracting the information contained in them, is a difficult task due to the vast diversity of ideas on YouTube. Identifying public opinion on war topics is crucial for offering insights to opposing sides based on popular opinion and emotions about the ongoing war. To address the gap, we build a model on YouTube comment sentiment analysis of the Hamas-Israel war to determine public opinion. In this study, we address the gaps by developing a deep learning-based approach for sentiment analysis. We have collected 24,360 comments from popular YouTube News Channels including BBC, WION, Aljazeera, and others about the Hamas-Israel War using YouTube API and Google spreadsheet and labeled them by linguistic experts into three classes: positive, negative, and neutral. Then, textual comments were preprocessed using natural language processing (NLP) techniques, and features were extracted using Word2vec, FastText, and GloVe. Moreover, we have used the SMOTE data balancing technique and used different data splits, but the 80/20 train-test split ratio has the highest accuracy. For classification model building, commonly used classification algorithms LSTM, Bi-LSTM, GRU, and Hybrid of CNN and Bi-LSTM were applied, and their performance is compared. As a result, the Hybrid of CNN and Bi-LSTM with Word2vec achieved the highest performance with 95.73% accuracy for comments classifications.

## Introduction

On October 7, Hamas launched a multipronged attack against Israel, targeting border villages and extending checkpoints around the Gaza Strip. The attack used armed rockets, expanded checkpoints, and helicopters to infiltrate towns and kidnap Israeli civilians, including children and the elderly^1^. Moreover, the Gaza conflict has led to widespread destruction and international debate, prompting sentiment analysis to extract information from users’ thoughts on social media, blogs, and online communities^2^. Israel and Hamas are engaged in a long-running conflict in the Levant, primarily centered on the Israeli occupation of the West Bank and Gaza Strip, Jerusalem's status, Israeli settlements, security, and Palestinian freedom^3^. Moreover, the conflict in Hamas emerged from the Zionist movement and the influx of Jewish settlers and immigrants, primarily driven by Arab residents' fear of displacement and land loss. Additionally, in 1917, Britain supported the Zionist movement, leading to tensions with Arabs after WWI. The Arab uprising in 1936 ended British support, resulting in Arab independence^1,4^.

Since the beginning of the November 2023 conflict, many civilians, primarily Palestinians, have died. Along with efforts to resolve the larger Hamas-Israeli conflict, many attempts have been made to resolve the conflict as part of the Israeli-Palestinian peace process^5^. Moreover, the Oslo Accords in 1993–95 aimed for a settlement between Israel and Hamas. The two-state solution, involving an independent Palestinian state, has been the focus of recent peace initiatives. The Quartet on the Middle East mediates negotiations, and the Palestinian side is divided between Hamas and Fatah^6^.

Organizations can enhance customer understanding through sentiment analysis, which categorizes emotions into anger, contempt, fear, happiness, sadness, and surprise^7^. Social media platforms such as Facebook, YouTube, and Twitter provide access to sentiment data, with YouTube being a significant subject of study for sentiment analysis Therefore, public opinion of the Russia-Ukraine conflict can be assessed by analyzing Reddit posts, with sentiments preprocessed and classified as neutral, positive, or negative^8,9^. Moreover, sentiment analysis offers valuable insights into conflicting viewpoints, aiding in peaceful resolutions. It aids in examining public opinion on social media platforms, aiding companies and content producers in content creation and marketing strategies. It also helps individuals identify problem areas and respond to negative comments^10^. Metadata, or comments, can accurately determine video popularity using computer linguistics, text mining, and sentiment analysis. YouTube comments provide valuable information, allowing for sentiment analysis in natural language processing^11^. Therefore, research on sentiment analysis of YouTube comments related to military events is limited, as current studies focus on different platforms and topics, making understanding public opinion challenging^12^.

The diverse opinions and emotions expressed in these comments are challenging to comprehend, as public opinion on war events can fluctuate rapidly due to public debates, official actions, or breaking news^13^. Managing hate speech and offensive remarks in war discussions on YouTube is crucial, requiring an understanding of user-generated content, privacy, and moral considerations, especially during wartime^14,15^. The unstructured nature of YouTube comments, the use of colloquial language, and the expression of a wide range of opinions and emotions present challenges for this task. Since the correlation between the front and back of a sequence cannot be described, traditional machine learning is ineffective in handling sequence learning. Sequence learning models such as recurrent neural networks (RNNs) which link nodes between hidden layers, enable deep learning algorithms to learn sequence features dynamically. RNNs, a type of deep learning technique, have demonstrated efficacy in precisely capturing these subtleties. Taking this into account, we suggested using deep learning algorithms to find YouTube comments about the Palestine-Israel War, since the findings will help Palestine and Israel find a peaceful solution to their conflict. Therefore, the structure of the remaining sections is shown as follows. Related works are revised in Section "Literature review". Section "Proposed model architecture" presents the proposed method and algorithm usage. Section "Experiment and results" presents the experiments and discussion. Section "Conclusion and recommendation" concludes the paper and outlines future work.

## Literature review

Since 2019, Israel has been facing a political crisis, with five wars between Israel and Hamas since 2006. The most recent war began in 2023 and continues as of today. Social media platforms such as YouTube have sparked extensive debate and discussion about the recent war. As such, we believe that sentiment analysis of YouTube comments about the Israel-Hamas War can reveal important information about the general public's perceptions and feelings about the conflict^16^. Moreover, social media's explosive growth in the last decade has provided a vast amount of data for users to mine, providing insights into their thoughts and emotions^17^. Social media platforms provide valuable insights into public attitudes, particularly on war-related issues, aiding in conflict resolution efforts^18^. Despite their precision and time-consuming nature, machine-learning algorithms are the foundation of sentiment analysis^16^.

Deep learning techniques, inspired by the brain's structural and autonomous learning ability, streamline computational model development and outperform standard machine learning in sentiment analysis, making them crucial for managing user-generated data^19^. Moreover, the unstructured nature of YouTube comments presents challenges for analysis, but recurrent neural networks (RNNs) excel in sequence learning, capturing subtle sentiments and enhancing their value for platforms such as YouTube and social media^11^.

Large volumes of data can be analyzed by deep learning algorithms, which can identify intricate relationships and patterns that conventional machine learning methods might overlook^20^. The context of the YouTube comments, including the author's location, demographics, and political affiliation, can also be analyzed using deep learning techniques. In this study, the researcher has successfully implemented a deep neural network with seven layers of movie review data. The proposed model achieves an accuracy of 91.18%, recall of 92.53%, F1-Score of 91.94%, and precision of 91.79%^21^.

Deep learning enhances the complexity of models by transferring data using multiple functions, allowing hierarchical representation through multiple levels of abstraction^22^. Additionally, this approach is inspired by the human brain and requires extensive training data and features, eliminating manual selection and allowing for efficient extraction of insights from large datasets^23,24^. Moreover, deep learning models, including deep belief networks (DBNs) convolutional neural networks (CNNs), recurrent neural networks (RNNs), and recursive neural networks, are highly effective at performing tasks such as text generation, vector representation, word estimation, sentence classification, modeling, and feature presentation^25^.

RNNs, including simple RNNs, LSTMs, and GRUs, are crucial for predictive tasks such as natural language understanding, speech synthesis, and recognition due to their ability to handle sequential data. Therefore, the proposed LSTM model classifies the sentiments with an accuracy of 85.04%. To experiment, the researcher collected a Twitter dataset from the Kaggle repository^26^. Therefore, their versatility makes them suitable for various data types, such as time series, voice, text, financial, audio, video, and weather analysis.

**Figure 1:**
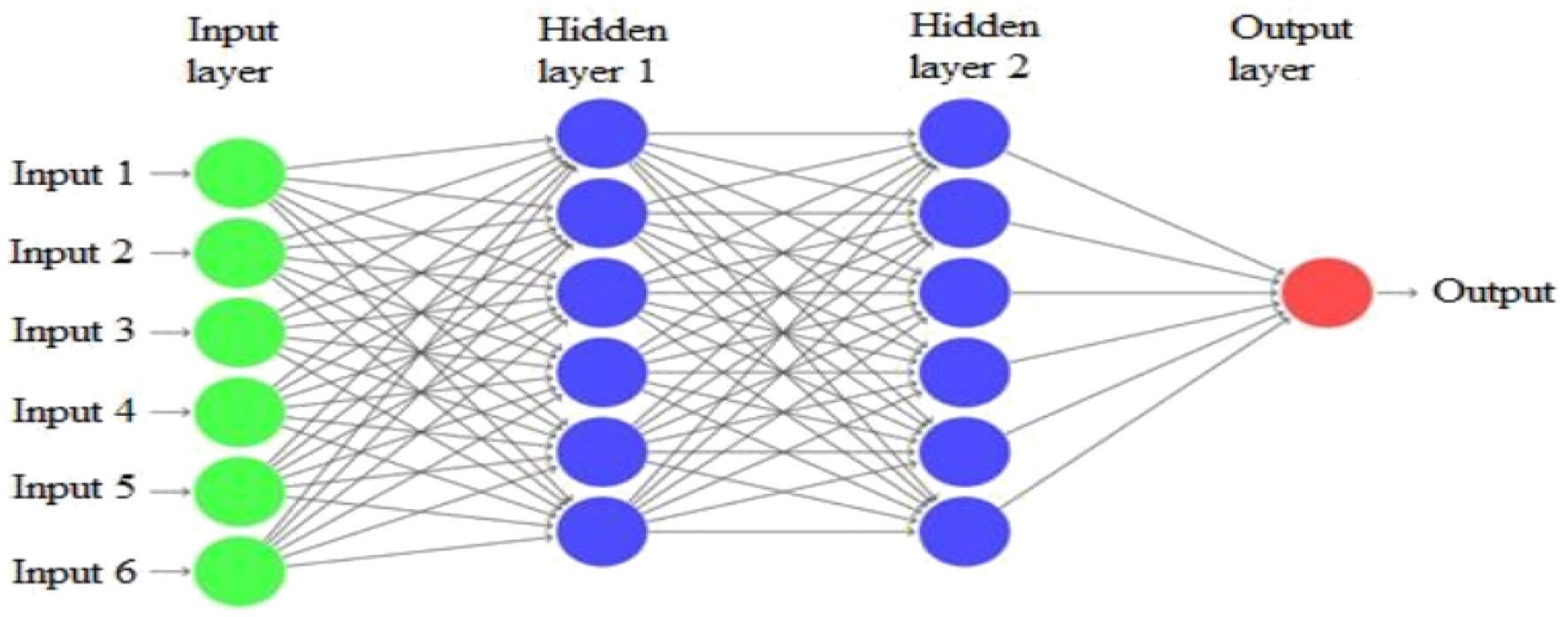
Recurrent neural network architecture adopted from Ref.^27^.

As described in Fig. 1, recurrent neural networks have many inputs, hidden layers, and output layers.

### Input layer

The first layer in a neural network is the input layer, which receives information, data, signals, or features from the outside world.

### Hidden layers

The layer responsible for extracting patterns associated with the analyzed system is the hidden layer in a neural network.

### Output layer

The output layer in a neural network generates the final network outputs based on the processing performed by the neurons in the previous layers. Recurrent neural networks (RNNs), commonly used for sentiment classification, often employ long short-term memory (LSTM), gated recurrent units (GRUs), and bidirectional long short-term memory (Bi-LSTM) units to process sequential data and produce final predictions.

LSTM networks enable RNNs to retain inputs over long periods by utilizing the skin of memory cells for computer memory. These cells function as gated units, selectively storing or discarding information based on assigned weights, which the algorithm learns over time. This adaptive mechanism allows LSTMs to discern the importance of data, enhancing their ability to retain crucial information for extended periods^28^.

The GRU (gated recurrent unit) is a variant of the LSTM unit that shares similar designs and performances under certain conditions. Like an LSTM network, a GRU controls information flow through gates. Although GRUs are newer and offer faster processing and lower memory usage, LSTM tends to be more reliable for datasets with longer sequences^29^. Moreover, they perform better than LSTM when handling smaller datasets^30^. Additionally, the study^31^ used to classify tweet sentiment is the convolutional neural network (CNN) and gated recurrent unit method (GRU). In this study, research stages include feature selection, feature expansion, preprocessing, and balancing with SMOTE. The highest accuracy value was obtained on the CNN-GRU model with an accuracy value of 95.69% value. Moreover, the LSTM neurons are split into two directions, one for forward states and the other for backward states, to form bidirectional LSTM networks^32^. Therefore, Bidirectional LSTM networks use input from past and future time frames to minimize delays but require additional steps for backpropagation over time due to the noninteracting nature of the two directional neurons^33^.

A study conducted by J. Liu^34^ investigated several approaches to address sentiment classification; in brief, the informal (Twitter) text multifilter CNN-Bi-LSTM outperforms existing models, achieving 85.1% accuracy. In addition, Ref.^35^ used BiLSTM for Twitter sentiment analysis and achieved better results than conventional neural networks. Moreover, according to Ref.^36^, in terms of accuracy and the F1-measure for sentiment analysis on social media, the Bi-LSTM-based training model performed better than the conventional LSTM model. The proposed model in Ref.^37^ employs a Bi-LSTM self-attention-based CNN model and a CNN for sentiment analysis of user reviews, achieving high classification accuracy and an F1 measure value. Moreover, Bidirectional recurrent neural networks combine only two independent GRUs. This layout ensures that networks have both backward and forward information about the array in each step. The bidirectional GRU processes inputs in two directions: past-to-future and future-to-past. This technique differs from the unidirectional one in that the GRU works backward, saving information from the future while combining two hidden states^38^. Moreover, the study uses a CNN layer and multi-layered bi-directional long-short-term memory (BiLSTM) to learn features from user reviews and feelings. The model achieves a test accuracy of 92.05% and a validation accuracy of 93.55%, proving effective against IMDB datasets^39^. Therefore, deep learning algorithms can provide insights into public opinion and sentiment in YouTube comments about the Hamas-Israel War, aiding in identifying a peaceful resolution.

## Proposed model architecture

**Figure 2:**
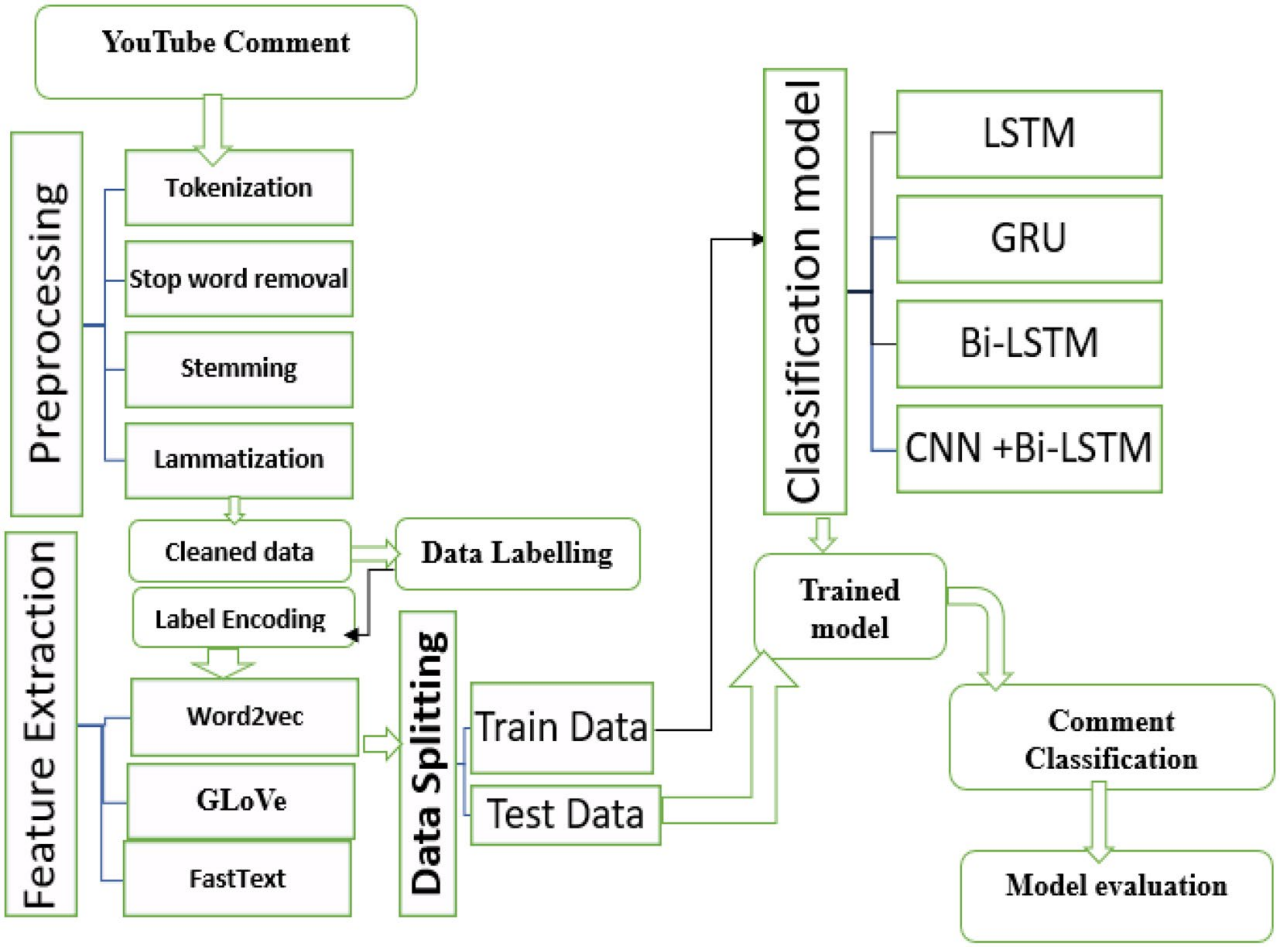
Proposed model architecture.

The general structure of the proposed model architectures is shown in Fig. 2. The proposed method is shown in Fig. 2 involves using LSTM, GRU, Bi-LSTM, and CNN-Bi-LSTM for sentiment analysis from YouTube comments.

### Dataset

The dataset was collected from various English News YouTube channels, such as CNN, Aljazeera, WION, BBC, and Reuters. We obtained a dataset from YouTube; we selected the popular channels and videos related to the Hamas-Israel war that had indicated dataset semantic relevance. Once selected the channel with the video, we used the YouTube API within a script, such as Google Apps Script, to fetch the desired pieces of comments on the video by adding a video ID on the Google Sheets. Therefore, the script makes requests to the API to retrieve video metadata about that video and store this comment in a dataset format, such as a CSV file or a Google Sheet. Therefore, we downloaded the prepared data from Google Sheets which consists of CNN of 2462, Aljazeera 4570, Reuters 6846, BBC of 2050, and WION of 8432, which we then annotated by linguistic experts as positive, negative, or neutral, respectively. As a result, Table 1 depicts the labeled dataset distribution per proposed class.

**Table 1:** Distributions of negative, positive, and neutral comments.

Class	Label	Number of data
Positive	1	7537
Negative	2	11,147
Neutral	0	5676
Total		24,360

### Preprocessing

To ensure that the data were ready to be trained by the deep learning models, several NLP techniques were applied. Models and algorithms must correctly predict outcomes. Preprocessing not only reduces the extracted feature space but also improves the classification accuracy^40^. The neat text and some NLP packages are used for text preprocessing. We used the following preprocessing techniques.

Tokenization is the process of separating raw data into sentence or word segments, each of which is referred to as a token. In this study, we employed the Natural Language Toolkit (NLTK) package to tokenize words. Tokenization is followed by lowering the casing, which is the process of turning each letter in the data into lowercase. This phase prevents the same word from being vectorized in several forms due to differences in writing styles.

The next step is to remove stop words. Stop words are words that relate to the most common words in a language and do not contribute much sense to a statement; thus, they can be removed without changing the sentence. Furthermore, stemming and lemmatization are the last NLP techniques used on the dataset. The two approaches are used to reduce a derived or inflected word to its root, base, or stem form. The distinction between stemming and lemmatization is that lemmatization assures that the root word (also known as a lemma) is part of the language.

Finally, we applied three different text vectorization techniques, FastText, Word2vec, and GloVe, to the cleaned dataset obtained after finishing the preprocessing steps. The process of converting preprocessed textual data to a format that the machine can understand is called word representation or text vectorization.

### Development tools and techniques

#### Python

Python is a high-level programming language that supports dynamic semantics, object-oriented programming, and interpreter functionality. Deep learning approaches for sentiment analysis are being tested in the Jupyter Notebook editor using Python programming.

#### NumPy

We utilized it to transform the text into numerical data to extract features and train and test our model.

#### Pandas

This is a popular Python library for importing and handling datasets.

#### TensorFlow

TensorFlow is commonly used for machine learning, deep learning, and numerical computations. It is an end-to-end platform that simplifies the development and deployment of deep learning models, and it serves as the backend for the Keras API.

#### Keras

Keras greatly simplifies deep learning tasks in Python. In this study, Keras was used to create, train, store, load, and perform all other necessary operations.

#### Matplotlib

Matplotlib is a plotting library. This study was used to visualize YouTube users’ trends from the proposed class perspectives and to visualize the model training history.

### Model training and evaluation

After the data were preprocessed, it was ready to be used as input for the deep learning algorithms. We obtained a better result when we used the 80/20 train-test split. The performance of the trained models was reduced with 70/30, 90/10, and another train-test split ratio. During the model process, the training dataset was divided into a training set and a validation set using a 0.10 (10%) validation split. Therefore train-validation split allows for monitoring of overfitting and underfitting during training. The training dataset is used as input for the LSTM, Bi-LSTM, GRU, and CNN-BiLSTM learning algorithms. Therefore, after the models are trained, their performance is validated using the testing dataset. The following metrics are used to evaluate the performance of each model.

- Recall: The true positives in a class are calculated from all the observations in the class. It is defined as true positive (TP)/TP + false positive (FP).
- Precision: This calculates the number of true positives out of all the input classes. It is defined as TP/TP + false negative (FN).
- F1: F1 was calculated based on precision and recall scores. It is defined as 2 * Precision (P) * Recall (R)/P + R.
- Accuracy: This calculates the number of true positives out of all the data points. It is defined as TP + true negative (TN)/TP + TN + FP + FN.

## Experiment and results

### Experiment

In this paper, classification is performed using deep learning algorithms, especially RNNs such as LSTM, GRU, Bi-LSTM, and Hybrid algorithms (CNN-Bi-LSTM). During model building, different parameters were tested, and the model with the smallest loss or error rate was selected. Therefore, we conducted different experiments using different deep-learning algorithms. As we mentioned in Table 1, there is a data imbalance, and we used techniques for balancing datasets such as oversampling minority classes, under-sampling majority classes, and more advanced methods like the synthetic minority over-sampling technique (SMOTE) for imbalanced datasets. Furthermore, dataset balancing occurs after preprocessing but before model training and evaluation^41^. As a result, balancing the dataset in deep learning leads to improved model performance and reduced overfitting. Therefore, the datasets have up-sampled the positive and neutral classes and down-sampled the negative class via the SMOTE sampling technique.

**Figure 3:**
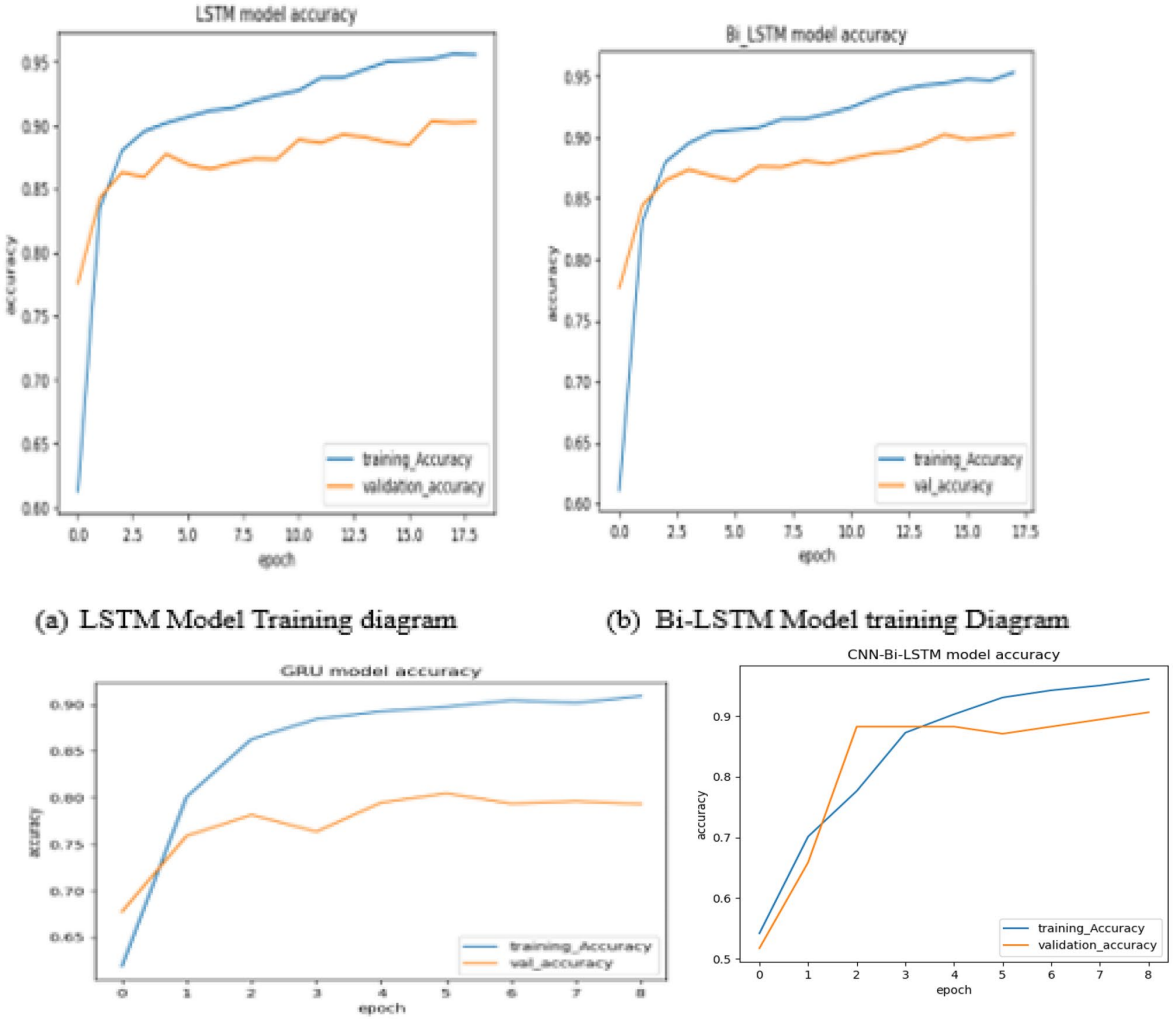
Different deep learning performance.

**Figure 4:**
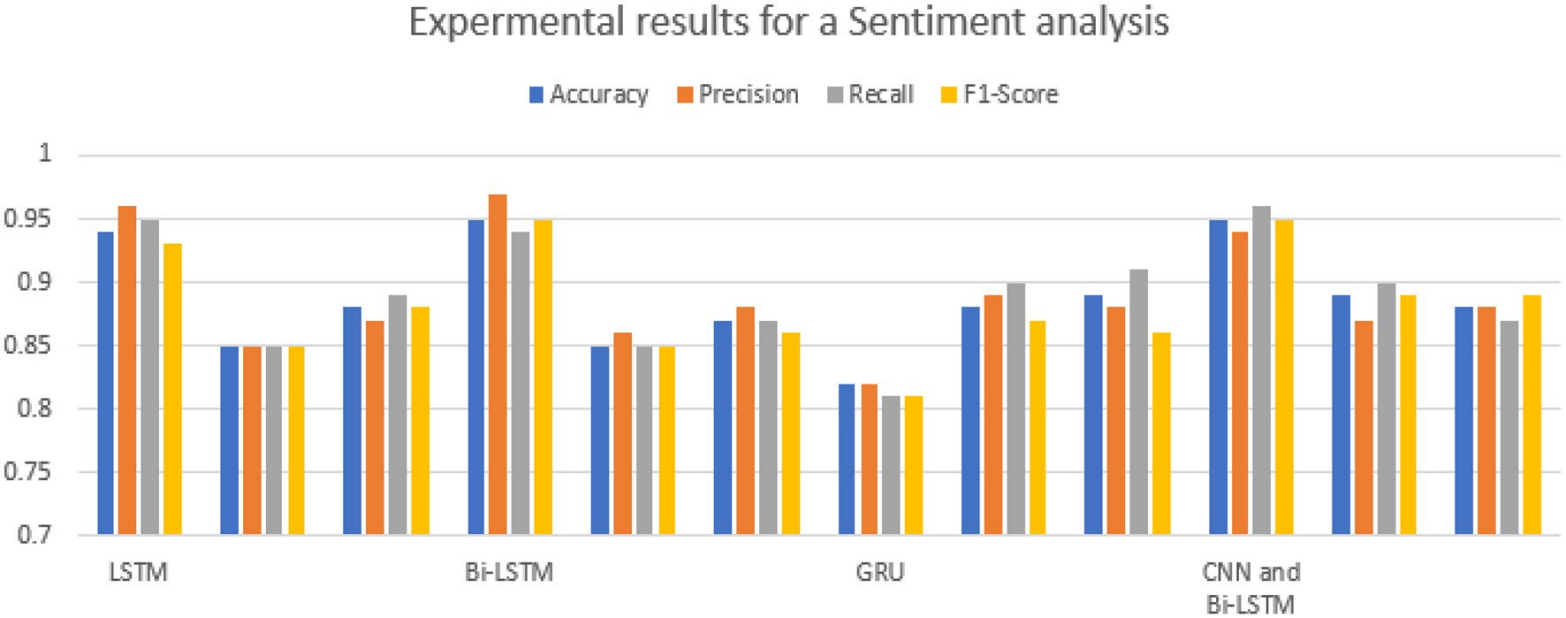
Visualization of the experimental dataset.

Next, the experiments were accompanied by changing different hyperparameters until we obtained a better-performing model in support of previous works. During the experimentation, we used techniques like Early-stopping, and Dropout to prevent overfitting. The models used in this experiment were LSTM, GRU, Bi-LSTM, and CNN-Bi-LSTM with Word2vec, GloVe, and FastText. As shown in Figs. 3 and 4, the accuracy of the results obtained using CNN-Bi-LSTM with Word2vec was far better, reaching 95.73%, and the accuracy of the results obtained using GRU, LSTM, and Bi-LSTM with similar parameters and feature extraction technique were 90.76%, 94.74%, and 95.33%, respectively.

To summarize the results obtained in this experiment, the results from CNN-Bi-LSTM achieved better results than those from the other Deep Learning as shown in the Fig. 3. The hyperparameters and the number of tests and training datasets used were the same for each model, even though the results obtained varied.

### Results and discussion

From the data visualization, we observed that the YouTube users had an opinion for the conflicted party to solve it peacefully. In this section, we also understand that so many users use YouTube to express their opinions related to wars. This shows that any conflicted country should view YouTube users for their decision. To categorize YouTube users’ opinions, we developed deep learning models, which include LSTM, GRU, Bi-LSTM, and Hybrid (CNN-Bi-LSTM). We trained the models using batch sizes of 128 and 64 with the Adam parameter optimizer. When we changed the size of the batch and parameter optimizer, our model performances showed little difference in training accuracy and test accuracy. Table 2 shows that the trained models with a batch size of 128 with 32 epoch size and Adam optimizer achieved better performances than those with a batch size of 64 during the experiments with 32 epoch size and Adam optimizer.

**Table 2:** Summary of the results of the proposed algorithm.

	LSTM			Bi-LSTM			GRU			CNN and Bi-LSTM		
	Word-2vec	Glove	FastText	Word-2vec	Glove	FastText	Word-2vec	Glove	FastText	Word-2vec	Glove	FastText
Accuracy	0.94	0.85	0.88	0.95	0.85	0.87	0.82	0.88	0.89	0.95	0.89	0.88
Precision	0.96	0.85	0.87	0.97	0.86	0.88	0.82	0.89	0.87	0.94	0.87	0.88
Recall	0.95	0.85	0.89	0.94	0.85	0.87	0.81	0.90	0.91	0.96	0.90	0.87
F1-score	0.93	0.85	0.88	0.95	0.85	0.86	0.81	0.87	0.89	0.95	0.89	0.89

As a result, testing of the model trained with a batch size of 128 and Adam optimizer was performed using training data, and we obtained a higher accuracy of 95.73% using CNN-Bi-LSTM with Word2vec to the other Deep Learning. The results of all the algorithms were good, and there was not much difference since both algorithms have better capabilities for sequential data. As we observed from the experimental results, the CNN-Bi-LSTM algorithm scored better than the GRU, LSTM, and Bi-LSTM algorithms. Finally, models were tested using the comment ‘**go-ahead for war Israel**’, and we obtained a negative sentiment. As described in the experimental procedure section, all the above-mentioned experiments were selected after conducting different experiments by changing different hyperparameters until we obtained a better-performing model.

## Conclusion and recommendation

### Conclusion

Social media websites are gaining very big popularity among people of different ages. Platforms such as Twitter, Facebook, YouTube, and Snapchat allow people to express their ideas, opinions, comments, and thoughts. Therefore, a huge amount of data is generated daily, and written text is one of the most common forms of the generated data. Business owners, decision-makers, and researchers are increasingly attracted by the valuable and massive amounts of data generated and stored on social media websites. Sentiment Analysis is a Natural Language Processing field that increasingly attracts researchers, government authorities, business owners, service providers, and companies to improve products, services, and research. Therefore, research on sentiment analysis of YouTube comments related to military events is limited, as current studies focus on different platforms and topics, making understanding public opinion challenging. As a result, we used deep learning techniques to design and develop a YouTube user sentiment analysis of the Hamas-Israel war. Therefore, we collected comments about the Hamas-Israel conflict from YouTube News channels. Next, significant NLP preprocessing operations are carried out to enhance our classification model and carry out an experiment on DL algorithms.

Therefore, LSTM, BiLSTM, GRU, and a hybrid of CNN and BiLSTM were built by tuning the parameters of the classifier. From this, we obtained an accuracy of 94.74% using LSTM, 95.33% using BiLSTM, 90.76% using GRU, and 95.73% using the hybrid of CNN and BiLSTM. Generally, the results of this paper show that the hybrid of bidirectional RNN(BiLSTM) and CNN has achieved better accuracy than the corresponding simple RNN and bidirectional algorithms. As a result, using a bidirectional RNN with a CNN classifier is more appropriate and recommended for the classification of YouTube comments used in this paper.

### Recommendation

Our model did not include sarcasm and thus classified sarcastic comments incorrectly. Furthermore, incorporating multimodal information, such as text, images, and user engagement metrics, into sentiment analysis models could provide a more holistic understanding of sentiment expression in war-related YouTube content. Therefore, we recommend that other researchers include sarcasm. Nowadays there are several social media platforms, but in this study, we collected the data from only the YouTube platform. Therefore, future researchers can include other social media platforms to maximize the number of participants. Social media users express their opinions using different languages, but the proposed study considers only English language texts. To solve this limitation future researchers can design bilingual or multilingual sentiment analysis models.

#### Practical limitations

Despite the vast amount of data available on YouTube, identifying and evaluating war-related comments can be difficult. Platform limits, as well as data bias, have the potential to compromise the dataset's trustworthiness and representativeness. Furthermore, the sheer volume of comments and the dynamic nature of online discourse may necessitate scalable and effective data collection and processing approaches.

#### Theoretical limitations

On a theoretical level, sentiment analysis innate subjectivity and context dependence pose considerable obstacles. Annotator bias and language ambiguity can all influence the sentiment labels assigned to YouTube comments, resulting in inconsistencies and uncertainties in the study.

## Data Availability

The datasets used and/or analyzed during the current study are available from the corresponding author upon reasonable request.
