# Author Correction: Sentiment analysis of the Hamas-Israel war on YouTube comments using deep learning

**DOI:** 10.1038/s41598-024-80821-4

**Published:** 2025-02-04

**Authors:** Ashagrew Liyih, Shegaw Anagaw, Minichel Yibeyin, Yitayal Tehone

**Affiliations:** 1 Department of Software Engineering, Debre Markos University, Debre Markos, Ethiopia; 2 Department of Business, University of Southeastern Norway, Drammen, Norway; 3 Department of Information Technology, Debre Markos University, Debre Markos, Ethiopia

Correction to: *Scientific Reports* 10.1038/s41598-024-63367-3, published online 13 June 2024

The original version of this Article contained errors in the References. Reference 9 was incorrectly given as Reference 1.

The correct Reference 1 is listed below:

Saturday, O. and Jazeera, A. Fact sheet: Israel and Palestine Conflict (October 2023), no. October, 9–12 (2023).

As a result, the subsequent References have been renumbered.

In the Introduction,

“The attack used armed rockets, expanded checkpoints, and helicopters to infiltrate towns and kidnap Israeli civilians, including children and the elderly^1^.”

now reads:

“The attack used armed rockets, expanded checkpoints, and helicopters to infiltrate towns and kidnap Israeli civilians, including children and the elderly^1^.”

 “Moreover, the conflict in Hamas emerged from the Zionist movement and the influx of Jewish settlers and immigrants, primarily driven by Arab residents’ fear of displacement and land loss^4^. Additionally, in 1917, Britain supported the Zionist movement, leading to tensions with Arabs after WWI. The Arab uprising in 1936 ended British support, resulting in Arab independence^5^.”

now reads:

“Moreover, the conflict in Hamas emerged from the Zionist movement and the influx of Jewish settlers and immigrants, primarily driven by Arab residents’ fear of displacement and land loss. Additionally, in 1917, Britain supported the Zionist movement, leading to tensions with Arabs after WWI. The Arab uprising in 1936 ended British support, resulting in Arab independence^1,4^.”

“Therefore, public opinion of the Russia-Ukraine conflict can be assessed by analyzing Reddit posts, with sentiments preprocessed and classified as neutral, positive, or negative^9^.”

now reads:

“Therefore, public opinion of the Russia-Ukraine conflict can be assessed by analyzing Reddit posts, with sentiments preprocessed and classified as neutral, positive, or negative^8,9^.”

The original Article has been corrected.

